# Effectiveness of a Self-Guided Digital Intervention for Mental Health and Psychological Well-Being in University Students: Pre- and Postintervention Study

**DOI:** 10.2196/69031

**Published:** 2025-08-19

**Authors:** Michela Nosè, Giulia Muriago, Giulia Turrini, Federico Tedeschi, Olga Forlani, Riccardo Sartori, Massimiliano Badino, Corrado Barbui

**Affiliations:** 1 WHO Collaborating Centre for Research and Training in Mental Health and Service Evaluation, Department of Neurosciences, Biomedicine, and Movement Sciences, Section of Psychiatry, University of Verona, Piazzale LA Scuro 10, Verona, 37134, Italy, 39 0458124440; 2 IT and Communications Management, University of Verona, Verona, Italy; 3 Department of Human Sciences, University of Verona, Verona, Italy

**Keywords:** college students, digital mental health, psychological well-being, mental health, university students, web-based intervention, implementation study

## Abstract

**Background:**

University students frequently face mental health challenges due to academic pressures, lifestyle changes, and developmental factors. Digital interventions, such as Doing What Matters in Times of Stress (DWM), a psychosocial e-mental health intervention developed by the World Health Organization (WHO), offer scalable approaches to address these issues. These data emerging from the literature provide the framework for the CAMPUS (Characterize and Address Mental health Problems in University Students) study aimed at supporting the mental health of students attending the University of Verona.

**Objective:**

This study aimed to assess the effectiveness and implementability of DWM as a psychological strategy for effective mental health prevention and promotion, as well as for reducing psychological symptoms and distress and improving well-being in university students.

**Methods:**

During the study period (October 2023-June 2024), we conducted a prospective hybrid type-1 nonrandomized follow-up study, with a pretest-posttest design. The study population consisted of students attending the University of Verona, who were recruited through university communication channels and participated via web-based platforms. Data were collected at baseline (T1) and after the intervention (T2) using an ad hoc sociodemographic information page and self-reported tools assessing psychological distress with the Kessler-10 (K-10), depressive symptoms with the Patient Health Questionnaire-9 (PHQ-9) depression scale, anxiety symptoms with the Generalized Anxiety Disorder-7 (GAD-7) scale, and psychological well-being with the WHO-5 Well-Being Index (WHO-5). In addition, at postintervention, the implementability was assessed. Statistical analyses included Wilcoxon matched pairs signed rank tests and logistic regression models to identify associated factors.

**Results:**

Out of 2296 interested students, 1498 (65.24%) completed all DWM sessions and assessments. At T1, students exhibited mild psychological distress, anxiety, and depressive symptoms with moderate well-being. Significant improvements were observed postintervention: the K-10 scores decreased from 22.41 (SD 6.54) to 19.86 (SD 5.96), the GAD-7 scale scores decreased from 8.27 (SD 4.31) to 6.57 (SD 3.76), and the PHQ-9 scores decreased from 8.28 (SD 7.73) to 6.75 (SD 4.37; all *P*<.001). The WHO-5 well-being scores increased from 11.73 (SD 4.65) to 13.26 (SD 4.68*; P*<.001). Satisfaction was high, with 90.72% (1359/1498) of participants agreeing or strongly agreeing on satisfaction, 77.37% (1159/1498) agreeing or strongly agreeing on appropriateness, and 94.99% (1423/1498) finding the program easy to use. No significant differences in clinical outcomes were associated with sociodemographic or baseline mental health variables.

**Conclusions:**

The DWM intervention demonstrated positive effects on students’ mental health, showing reductions in distress, anxiety, and depressive symptoms, alongside improved well-being. The program’s high levels of acceptability, appropriateness, and feasibility highlight its potential for broader application as a digital mental health strategy for university students.

## Introduction

### Background

Life at university provides important opportunities for personal growth; however, this developmental phase also coincides with the peak period of risk for the onset of mental disorders, including anxiety, depression, substance abuse, self-harm, and suicidal behavior [1]. In addition, specific university lifestyle factors, including impaired sleep and academic and financial stress [2], are known to exacerbate psychological distress in students. In the World Mental Health Surveys, a set of large-scale cross-national community epidemiological surveys, the 12-month prevalence of any mental disorder among university students was approximately 20% [3]. In 2018, the World Health Organization (WHO) reported that approximately one-third of first-year university students from 19 universities (13,948 respondents) across 8 countries (Australia, Belgium, Germany, Mexico, Northern Ireland, South Africa, Spain, and United States) screened positive for at least 1 common *Diagnostic and Statistical Manual of Mental Disorders, Fourth Edition (DSM-IV) *anxiety, mood, or substance disorder [4]. Similar results have been found in another international study conducted in 12 countries in Europe, Latin and North America, and Australia, with 48% of students presenting clinically relevant depressive symptoms [5]. More recent data from the World Mental Health International College Student initiative, which included >70,000 first-year university students from 18 countries, reported a significantly higher 12-month prevalence of any mental disorder, estimated at 57.4% [6]. Furthermore, due to the recent COVID-19 pandemic, an increase in the prevalence of mental health problems among university students has been reported, which may have exacerbated this pressing issue [7-9]. It was also found that students with mental disorders and psychological distress showed a dropout rate 2.5 times higher than that of matched controls [4]. As a result, university students have been identified as a population group considered vulnerable and often experiencing significant barriers to accessing psychological treatment [4]. University psychological counseling services are not always available, and even when they are, they have limited impact because of their isolation from the general health care system, the heterogeneity of the interventions offered, and the difficulties in managing the growing demand [10].

Digital psychological interventions are emerging as a promising solution for supporting mental health among university students [11-13]. Recent systematic reviews and meta-analyses [11,13,14] confirm the potential of these interventions to address mental health challenges in this population. Digital interventions provide an effective alternative in overcoming the barriers and challenges that university students often face when seeking mental health assistance. Given the positive effects on psychological well-being demonstrated in numerous studies, the literature increasingly supports the development of evidence-based digital interventions tailored to improve psychological well-being among groups considered vulnerable such as university students.

e-Mental health solutions, including smartphone apps, are a new type of tool widening the means through which persons in need can access psychosocial support [15]. Their use could potentially improve access to mental health support, as they are, in principle, widely available and inexpensive. Smartphone apps may be particularly appealing due to their anonymity, portability, and ease of access. Hence, the WHO has developed several psychosocial e-mental health interventions, including Doing What Matters in Times of Stress (DWM) [16], a stress management guide that provides practical information and skills to help people cope with stressful situations based on the principles of acceptance and commitment therapy. Evidence from randomized trials has demonstrated the effectiveness of DWM in various populations considered vulnerable, such as migrants or health care workers [17-19]; this suggests that DWM is particularly suitable for people exposed to a wide variety of adversities, helping them address diverse challenges and manage stress.

### Objectives

These data emerging from the international literature provide the framework for the CAMPUS (Characterize and Address Mental health Problems in University Students) study, which is aimed at students at the University of Verona and has been created in response to the growing need to support the mental well-being of university students. The principal objectives of the study are to evaluate the effectiveness and implementability of self-guided DWM as a psychological strategy for effective mental health prevention and promotion, as well as for reducing psychological symptoms and distress and improving well-being in university students. Secondary objectives of the project include assessing whether there are groups of students who could benefit most from the intervention and identifying factors associated with its implementability and effectiveness.

## Methods

### Study Design

The project was implemented during the 2023/2024 academic year: first, during the first academic semester (October 2023 to January 2024) and then in the second semester (from March 2024 to June 2024). The DWM intervention was offered to students both as a Teaching and Learning Centre course, that is, as a 1-credit educational activity, and as a service provided by the University of Verona. During the study period, we conducted a prospective hybrid type-1 nonrandomized follow-up study, as described by Curran et al [20] with a pretest-posttest design, where the first assessment (T1) was completed before gaining access to the DWM intervention, while the second assessment was conducted 1 week after the end of the intervention (T2) [21,22]. A detailed protocol for this study was registered in Open Sciences Framework [23].

### Target Population

The study population consisted of students at the University of Verona, which enrolled 25,756 students during the 2023/2024 academic year. Students were recruited through short announcements about the study in class as well as through flyers, emails, and other communication strategies.

### Data Collection and Study Procedures

Before enrollment, the students were informed online about the nature of the study and the intervention, and then, they chose whether to participate in the study. It was specified that they could withdraw from the study at any time and that the choice to participate, decline, or withdraw had no impact on their academic career. A web-based informed consent was electronically signed by all students who decided to participate. Participants registered on the Moodle platform (Moodle Pty Ltd), an e-learning platform used by the University of Verona for its students, and had access to LimeSurvey (LimeSurvey GmbH), the software used in this study to collect pre- and postintervention data. The pre- and postintervention assessments consisted of the collection of ad hoc sociodemographic information (age, gender, living condition, and characteristics of the course of study) and information on psychological condition, which was collected through 4 self-administered questionnaires assessing psychological distress with the Kessler-10 (K-10) [24], depressive symptoms with the Patient Health Questionnaire-9 (PHQ-9) depression scale[25], anxiety symptoms with the Generalized Anxiety Disorder-7 (GAD-7) scale [26], and psychological well-being with the WHO-5 Well-Being Index (WHO-5) [27]. In addition, at postintervention, the acceptability, appropriateness, and feasibility of the intervention were assessed using the adapted versions of the Acceptability of Intervention Measure (AIM), the Intervention Appropriateness Measure (IAM), and the Feasibility of Intervention Measure (FIM) [28]. The preintervention assessment (T1) was completed by students before gaining access to the DWM intervention, while the postintervention assessment was completed after the end of the intervention (T2). The data collected through LimeSurvey were stored safely in a password-protected CSV file for further analysis. After completing the assessment at T1, participants were able to access the DWM intervention on the Moodle platform. They received email reminders to complete the assessments. We kept track of every participant’s activity using the intervention metadata. The research team was available to provide support for technical issues, remote process monitoring, overcoming potential barriers, and referral to health services in case of need.

### Inclusion Criteria

Considering that study participation was limited to students enrolled at the University of Verona, participants were aged ≥18 years. Students who electronically signed the informed consent form before entering the study were enrolled. Only students attending the 5 DWM sessions were included in the evaluation. No exclusion criteria were applied.

### Intervention: DWM

DWM is an illustrated self-help guide developed by the WHO to support stress management and coping. The guide aims to equip people with practical skills to help cope with stress. For the purposes of this study, this self-help guide was adapted for digital delivery on the Moodle platform. The DWM intervention consists of an e-book divided into 5 monographic chapters covering 5 acceptance- and mindfulness-based strategies for managing stress. The strategies are as follows: grounding, unhooking, acting on your values, being kind, and making room. The chapters include audio recordings with different practices and exercises designed to help identify barriers and facilitators to practicing, as well as triggers that exacerbate stress responses. For our project, DWM has been adapted for digital delivery on the Moodle platform, allowing for self-guided, asynchronous access. Participants registered on the Moodle platform were given access to written and recorded materials for each of the 5 core component skills and activities of DWM and were asked to complete 1 chapter per week for a total of 5 weeks of intervention, as suggested by the DWM protocol. Participants could engage with the materials at their own pace and in their preferred setting, such as at home or on campus, using any device with internet access.

### Effectiveness Measures

#### Psychological Distress

The K-10 is a 10-item self-report questionnaire designed to broadly screen for psychological distress experienced in the past 30 days. Its items are rated on a 5-point Likert scale, ranging from “none of the time” to “all of the time.” The K-10 has good psychometric properties and has strong discriminatory power to distinguish *DSM-IV* cases from noncases [24].

#### Depression and Anxiety Symptoms

The Patient Health Questionnaire Anxiety and Depression Scale is a 16-item self-reported instrument that combines PHQ-9 and GAD-7 into a composite measure of depression and anxiety [29]. Respondents are asked how much each symptom has bothered them over the past 2 weeks, with response options of “not at all,” “several days,” “more than half the days,” and “nearly every day” scored as 0, 1, 2, and 3, respectively. The scale can range from 0 to 27 in the case of PHQ-9 and from 0 to 21 in the case of GAD-7, with higher scores indicating higher levels of depression and anxiety symptoms.

#### Psychological Well-Being

The WHO-5 is a 5-item questionnaire measuring current psychological well-being and quality of life, rather than psychopathology. Scores range from 0 to 25, and the scale has demonstrated sensitivity to change in well-being and is available in numerous languages [27].

All the measures were adapted to be fulfilled in LimeSurvey.

### Implementability Measures

Implementability measures include acceptability, appropriateness, and feasibility. Quantitative data on participants’ points of view on the implementability of DWM were gathered. For this purpose, adapted versions of the AIM, IAM, and FIM [28] were administered during the T2 assessment. In particular, 4 items were selected: 2 for acceptability (“The ‘Doing What Matters in Times of Stress’ program has satisfied me” and “The program ‘Doing What Matters in Times of Stress’ has interested me”), 1 for appropriateness (“The ‘Doing What Matters in Times of Stress’ program seemed appropriate for my needs”), and 1 for feasibility (“The program ‘Doing What Matters in Times of Stress’ seemed easy for me to use”). Scale values range from 1 to 5. Norms and cut-off scores for interpretation are not yet available; however, higher scores indicate greater acceptability, appropriateness, or feasibility [30].

### Statistical Analysis

Continuous variables were expressed as means and SDs, while categorical variables were expressed as absolute numbers and percentages. Continuous variables were also categorized; for age, the groups were as follows: ≤20, 21‐23, 24‐25, 26‐30, and >30 years. As for clinical variables, presence of psychological distress was assessed through K-10 values ≥16 [24]; GAD-7 was categorized as follows: absent or minimal anxiety: 0‐4, mild anxiety: 5‐9, moderate anxiety: 10‐14, and severe anxiety: 15‐21; PHQ-9 was grouped as follows: absence of depression: 0‐4, subthreshold depression: 5‐9, mild major depression: 10‐14, moderate major depression: 15‐19, and severe major depression: 20‐27; and finally, WHO-5 was grouped into low (1-8), medium (9-16), and high (17-25) levels of well-being.

In the case of clinical outcomes, a Wilcoxon matched pairs signed rank test was performed to compare baseline and posttreatment distributions. The clinical outcomes at T2 were regressed on age, gender, year of attendance (first 3 y, y 4‐6 or students awaiting graduation, or extended enrollment), housing status (owned, rented, or other), and living situation (with parents, flatmates, alone, other), controlling for the values of the scale at T1 and the area of study. The Seemingly Unrelated Regression model [31] was implemented to perform a global test on all the predictors of interest and, in case of statistical significance, a joint test for each variable; only in case of statistical significance of such tests, the *P* values from the independent regressions on each clinical outcome were considered.

The implementability outcomes were dichotomized as 5 (“completely agree”) versus 1‐4. The implementability outcomes were regressed with logistic regressions on the clinical outcomes at baseline and the same predictors used for the previously described regressions on clinical outcomes. Four logistic regressions were performed simultaneously; in particular, a seemingly unrelated estimation [32] model was implemented to perform a global test on all the predictors of interest and, in case of statistical significance, a joint test for the sociodemographic and the clinical variables separately and finally, in case of further statistical significance, for each variable; only in case of statistical significance of such tests, the *P* values from the independent logistic regressions were considered.

Multiple imputations were adopted to address the issue of missing data in all the variables included in the abovementioned models, using the *ice* routine in Stata [33,34]. To avoid out-of-range imputed values, the implementability measures (as single-item scores) were treated as ordered categorical variables during imputation, while a lower bound of 18 years was set for age. The number of imputed samples was determined by following the rule of thumb suggested by White et al [35], that is, “at least equal to the percentage of incomplete cases.” This number was then rounded up to the nearest multiple of 10.

A power calculation based on the K10 global score was conducted. Assuming an SD both at baseline and posttreatment of approximately 7.5 [36] and a correlation between baseline and posttreatment values of approximately 0.2, we hypothesized an SD of the difference of 9.5. The minimum sample size required to get a 95% CI with a maximum width of 2 is 350 (power calculations performed using the PASS software (NCSS, LLC) [37]). Assuming an attrition rate of 30%, the number of participants to recruit for the implementation phase was set to 500.

Statistical analyses were implemented with the software Stata 18 (StataCorp LLC) [38].

### Ethical Considerations

This study received ethics approval by the Institutional Review Board of the University of Verona (registration number 2023-UNVRCLE-0362987). Only participants who provided informed consent were included in this study. Data were collected anonymously and securely stored by the research team in a locked file. The participants answered the questions on a completely voluntary basis, and no compensation was provided.

## Results

### Sociodemographic and Clinical Characteristics at Baseline

**Figure 1.**
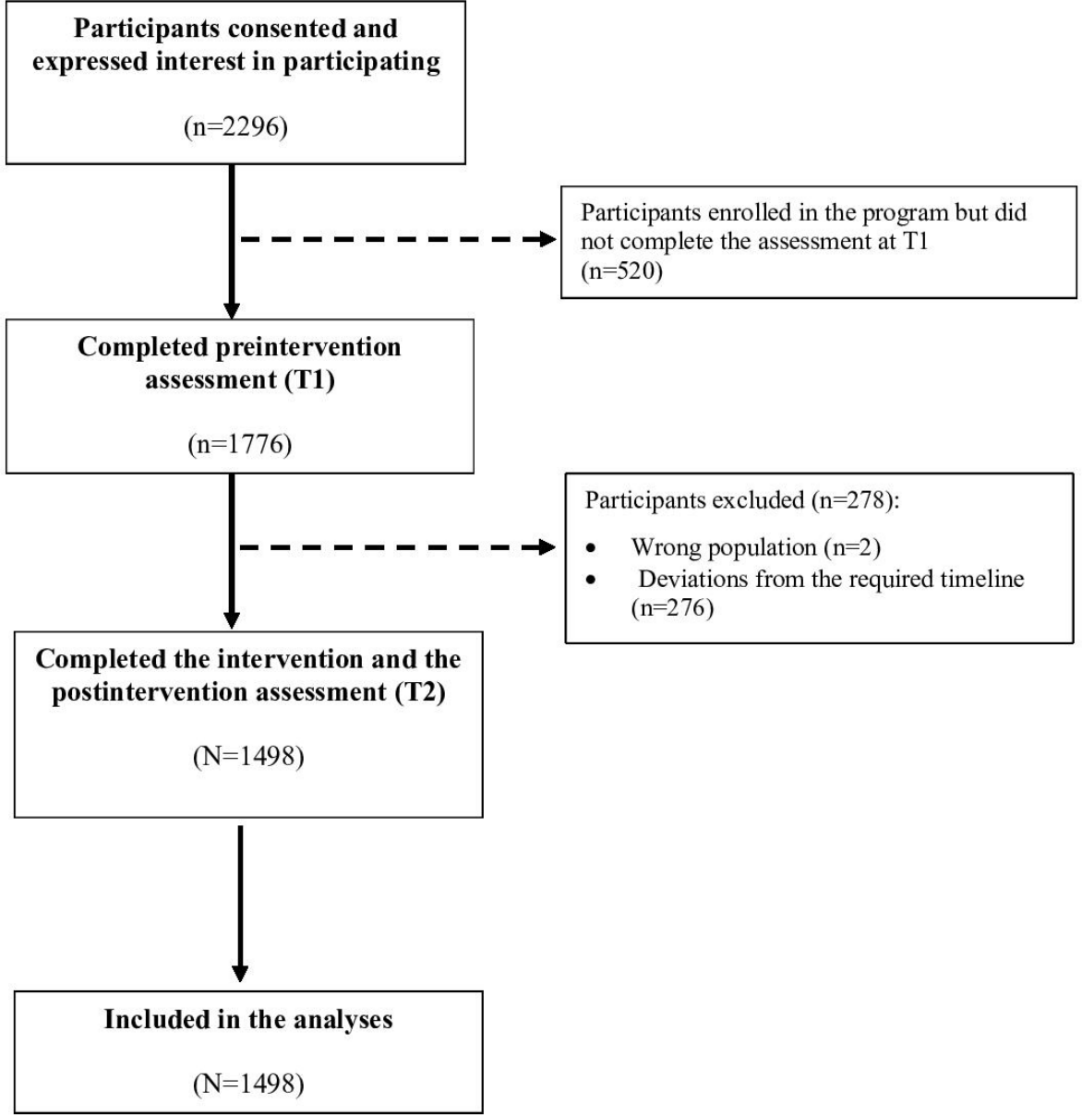
Flowchart detailing participant progress through the study. T1: preintervention; T2: postintervention.

A total of 2296 students at the University of Verona expressed interest in the course; of these, 1498 (65.24%) were included in the analyses because they attended all the DWM sessions and completed the questionnaires at T1 and T2. The participant flow diagram provides a detailed outline of dropout numbers and reasons for exclusion from analysis at each study stage (Figure 1). Most of the students (1149/1498, 76.7%) recruited were women, with an average age of 23.5 (SD 5.74) years. Almost half (679/1498, 45.32%) were studying medicine and surgery or economics, and a significant proportion (1017/1498, 67.89%) were living with their families, while only 18.42% (276/1498) were living with flatmates (Table 1).

**Table 1.** Sociodemographic characteristics (N=1498).

		Participants
Gender, n (%)		
	Female	1149 (76.70)
	Male	347 (23.16)
	Other	2 (0.13)
Age (years), mean (SD; range)		23.50 (5.74; 18‐64)
Age (years), n (%)		
	≤20	305 (20.36)
	21‐23	769 (51.34)
	24‐25	153 (10.21)
	26‐30	105 (7.01)
	>30	118 (7.88)
	Missing	48 (3.20)
Area of study, n (%)		
	Economics	329 (21.96)
	Education, philosophy, and social work	198 (13.22)
	Law	105 (7.01)
	Literature, arts, and communication studies	109 (7.28)
	Foreign languages and literatures	127 (8.48)
	Medicine and surgery	350 (23.36)
	Sciences and engineering	190 (12.68)
	Sport science	82 (5.47)
	Missing	8 (0.53)
Year of attendance, n (%)		
	First	296 (19.76)
	Second	453 (30.24)
	Third	582 (38.85)
	Fourth	72 (4.81)
	Fifth	63 (4.21)
	Sixth	9 (0,60)
	Students awaiting graduation	4 (0.27)
	Extended enrollment	17 (1.13)
	Missing	2 (0.13)
Housing status, n (%)		
	Owned	1036 (69.16)
	Rented	417 (27.84)
	Other	32 (2.14)
	Missing	13 (0.87)
Living situation, n (%)		
	Parents	1017 (67.89)
	Flatmates	276 (18.42)
	Partner	93 (6.21)
	Partner and children	46 (3.07)
	Alone	50 (3.34)
	Other	14 (0.93)
	Missing	2 (0.13)

**Figure 2.**
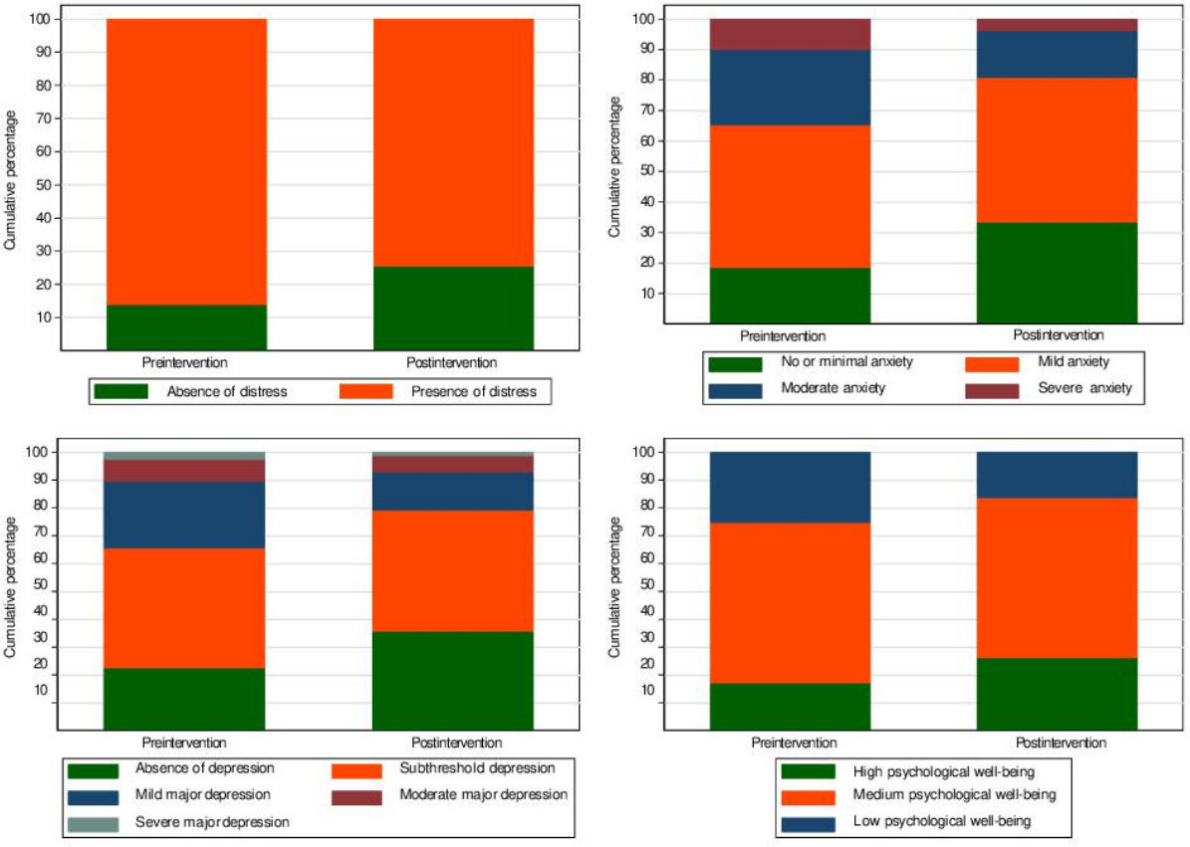
Clinical outcomes pre– and post–Doing What Matters in Times of Stress intervention.

As reported in Table 2 and Figure 2, at baseline (T1), participants, on average, showed mild levels of psychological distress, anxiety, and depressive symptoms, coupled with medium to low levels of psychological well-being.

**Table 2.** Clinical variables (N=1498).

		T1		T2		*P* values^a^
K-10^b^_TOT^c^, n (%)						<.001
	Absence of psychological distress	208 (13.89)		382 (25.5)		
	Presence of psychological distress	1290 (86.11)		1116 (74.5)		
K-10_TOT, mean (SD)		22.41 (6.54)		19.86 (5.96)		—^d^
GAD-7^e^_TOT, n (%)						<.001
	Absent or minimal anxiety	277 (18.49)		500 (33.38)		
	Mild anxiety	701 (46.8)		711 (47.46)		
	Moderate anxiety	368 (24.57)		228 (15.22)		
	Severe anxiety	152 (10.15)		59 (3.94)		
GAD-7_TOT, mean (SD)		8.27 (4.31)		6.57 (3.76)		—
PHQ-9^f^_TOT, n (%)						<.001
	Absence of depression	337 (22.5)		535 (35.71)		
	Subthreshold depression	647 (43.19)		652 (43.52)		
	Mild major depression	356 (23.77)		205 (13.68)		
	Moderate major depression	121 (8.08)		89 (5.94)		
	Severe major depression	37 (2.47)		17 (1.13)		
PHQ-9_TOT, mean (SD)		8.28 (4.73)		6.75 (4.37)		—
WHO-5^g^_TOT, n (%)						<.001
	Low psychological well-being (1-8)	378 (25.23)		246 (16.42)		
	Medium psychological well-being (9-16)	865 (57.74)		859 (57.34)		
	High psychological well-being (17-25)	255 (17.02)		393 (26.23)		
WHO-5_TOT, mean (SD)		11.73 (4.65)		13.26 (4.68)		—

a Wilcoxon matched pairs signed rank test.

b K-10: Kessler-10.

c TOT: total score.

d Not applicable.

e GAD-7: Generalized Anxiety Disorder-7.

f PHQ-9: Patient Health Questionnaire-9.

g WHO-5: World Health Organization-5 Well-Being Index.

### Effectiveness of DWM

After completing the DWM intervention (T2), we observed significant improvements across all measured outcomes, with a reduction in psychological symptoms, anxiety, and depressive symptoms and an increase in general psychological well-being. The mean score for psychological distress (K-10) decreased from 22.41 (SD 6.54) at baseline to 19.86 (SD 5.96) at postintervention, indicating a significant reduction in distress levels (*P*<.001). At T1, 86.11% (1290/1498) of participants showed signs of psychological distress, which decreased to 74.5% (1116/1498) at T2. Mean anxiety scores (GAD-7) decreased from 8.27 (SD 4.31) at baseline to 6.57 (SD 3.76) at postintervention (*P*<.001). The proportion of students with moderate or severe anxiety dropped from 34.71% (520/1498) at T1 to 19.16% (287/1498) at T2. Depression scores (PHQ-9) also improved, with a mean score reduction from 8.28 (SD 7.73) at baseline to 6.75 (SD 4.37) at postintervention (*P*<.001). The proportion of students with mild to severe depression dropped from 34.31% (514/1498) to 20.76% (311/1498). The mean score for psychological well-being (WHO-5) increased from 11.73 (SD 4.65) at T1 to 13.26 (SD 4.68) at T2 (*P*<.001), reflecting a significant enhancement in students’ well-being. The percentage of students reporting high well-being increased from 17.02% (255/1498) at T1 to 26.23% (393/1498) at T2.

### Implementability of DWM

In relation to the implementability outcomes, 90.72% (1359/1498) of participants either agreed or completely agreed that the DWM program satisfied them, 77.37% (1159/1498) felt that the program was appropriate for their needs, and 94.99% (1423/1498) agreed or completely agreed that the program was easy to use, highlighting its practicality for students (Table 3).

**Table 3.** Measures of satisfaction of Doing What Matters in Times of Stress (N=1498).

		Completely disagree, n (%)	Disagree, n (%)	Neither agree nor disagree, n (%)	Agree, n (%)	Completely agree, n (%)	Missing, n (%)
Acceptability measures							
	The program has satisfied me	6 (0.40)	16 (1.07)	116 (7.74)	954 (63.68)	405 (27.04)	1 (0.07)
	The program has interested me	6 (0.40)	9 (0.60)	77 (5.14)	806 (53.81)	599 (39.99)	1 (0.07)
Appropriateness measure							
	The program seemed appropriate for my needs	9 (0.60)	47 (3.14)	282 (18.83)	809 (54.01)	350 (23.36)	1 (0.07)
Feasibility measure							
	The program seemed easy for me to use	8 (0.53)	5 (0.33)	61 (4.07)	550 (36.72)	873 (58.28)	1 (0.07)

### Factors Associated With Clinical and Implementability Outcomes

Given that 4.81% (72/1498) of observations had missing values, only 10 imputations were performed.

Regression analyses revealed no significant differences in clinical response to the intervention based on sociodemographic factors, baseline mental health status, or type of area of study (*F*_36, 5913.8_=1.03; *P*=.43).

In the case of implementability outcomes, the fact that both nonbinary students (gender: other) chose the “completely agree” option to all item questions made their inclusion in the logistic regression model impossible because of the issue of quasi-separation. The items of interest were globally statistically significant (*F*_48, 8__,400,000_=2.48; *P*<.001). Both sociodemographic (*F*_32, 2,7__00,000_=1.62; *P=*.02) and clinical (*F*_16, 2__90,000,000_=4.31; *P*<.001) variables overcame the significance threshold.

Looking at single predictors, gender (*F*_4, 1,2__00,000,000_=3.18; *P*=.01) and WHO-5 (*F*_4, 5__5,000,000_=7.98; *P*<.001) turned out to be statistically significant, while, looking at single regressions, in both cases, significance was found for the acceptability and appropriateness outcomes. In particular, the odds ratios for female gender and WHO-5 scores were as follows: 1.506 (*P*=.009) and 1.107 (*P*<.001) for satisfaction, 1.625 (*P*=.001) and 1.044 (*P*=.008) for interest, and 1.466 (*P*=.02) and 1.085 (*P*<.001) for appropriateness, respectively. Such results highlight that, ceteris paribus, being female and having a higher WHO score at baseline corresponded to a significantly higher probability to “completely agree” with the acceptability and appropriateness items.

Figure S1 in Multimedia Appendix 1 shows, for such items, the probability of choosing the “completely agree” option for each WHO score, with its CI, for males and females separately, in case all the other variables were kept equal.

## Discussion

### Principal Findings

This study evaluated the effectiveness and implementability of WHO’s DWM intervention as a digital strategy for mental health promotion and prevention, specifically aimed at reducing psychological symptoms and distress among university students. The findings provide strong evidence supporting DWM’s beneficial impact as an applicable intervention to enhance psychological well-being in this population. Notably, there was a consistent improvement in well-being, as measured by the WHO-5, with significant reductions in distress, depression, and anxiety, regardless of students’ demographics or baseline mental health. Our results are in line with the evidence from the literature, which has highlighted the effectiveness of digital mental health interventions, particularly web-based or computer-delivered interventions, in decreasing depression, anxiety, stress, and eating disorder symptoms in the student population [12]. Delivered digitally, DWM addresses common barriers such as stigma, time constraints, and accessibility, which often discourage students from seeking in-person mental health support. University students have reported that digital mental health services are convenient and easy to use [39], helping them overcome challenges such as scheduling conflicts, waitlists, inaccessibility, and added expenses [40]; in addition, these services reduce the stigma associated with seeking mental health care by fostering a sense of ownership over one’s mental health and encouraging help-seeking behavior [40]. DWM is founded as a promising tool that can help overcome all these structural and psychological barriers. This is also demonstrated by the fact that most students reported satisfaction with the proposed intervention and considered it appropriate and easy to use, indicating that DWM has strong implementability [13].

The study also highlights that no sociodemographic or clinical characteristic at baseline predicted a different response to DWM. Regression analysis showed no significant differences in outcome based on sociodemographic factors, initial mental health status, or field of study, suggesting DWM’s equal effectiveness across diverse groups, such as age, gender, and academic discipline. This universality is significant, as it confirms DWM’s suitability as a comprehensive mental health strategy, effective across a wide-reaching population without needing customization. In line with prior studies on digital interventions [10,12], these results suggest DWM’s effectiveness for diverse populations due to its accessibility and adaptability to individual needs, suggesting DWM’s scalability as a valuable tool for universities in addressing the mental health needs of all students inclusively.

In university settings, where students encounter diverse stressors and may lack access to specialized support, a broad, generalizable approach such as self-guided DWM can meet the mental health needs of the entire student body, fostering resilience and well-being across all demographics. Given its asynchronous format and ease of access through digital platforms, it can be easily integrated into existing university mental health programs, allowing students to engage with the material at their own pace. Moreover, it can be embedded within broader student well-being initiatives, complementing traditional services such as counseling, workshops, and peer support, while also serving as an effective early intervention tool accessible to all students. The feasibility of DWM as part of a universal mental health strategy for higher education is particularly strong, offering an inclusive academic environment that supports the psychological resilience of all students. Unlike interventions requiring adaptation for specific subgroups, DWM demonstrates benefits for all students, regardless of their initial levels of psychological distress. This universality is a significant strength, as university students experience a broad range of mental health challenges, though not all may seek or qualify for specialized support [13].

These findings suggest several avenues for future research. First, future research should explore the effectiveness of DWM within this specific population through randomized trials, which could yield more robust, population-specific evidence and targeted results. Moreover, further efforts could explore the specific mechanisms by which DWM reduces distress and enhances well-being, examining components such as grounding exercises or value-driven actions to pinpoint elements that most benefit student outcomes. Second, implementing DWM within a broader digital mental health framework, including mobile apps and web-based support communities, could broaden its impact by catering to diverse student mental health needs.

Finally, this study’s results have particular relevance for low-income settings, where university students face significant barriers to mental health support but frequently have internet access. Digital interventions such as DWM, being low cost, scalable, and remotely accessible, offer a promising approach to closing mental health service gaps in these settings [12]. The adaptability of DWM to different demographic and psychological profiles highlights its suitability across various cultural and socioeconomic backgrounds. In low-resource settings, where mental health infrastructure is limited, DWM could play a crucial role in promoting well-being, reducing distress, and supporting academic success. This aligns with global health priorities advocating accessible, evidence-based digital solutions for mental health, particularly in underserved populations. The widespread availability of smartphones and internet among young people in low-income countries makes DWM an ideal candidate for integration into university programs, potentially fostering resilience and reducing disparities in mental health. Future research could focus on implementing and culturally adapting DWM in these settings, investigating factors such as digital literacy, internet stability, and local perceptions of digital mental health to further optimize its impact.

### Limitations

It is important to acknowledge that this study has some limitations. First, the recruited sample, although large, may not be entirely representative of the broader university student population. The majority of participants (1149/1498, 76.7%) were women, which, although partially reflective of the overall student population at the University of Verona, still indicates a degree of gender overrepresentation that may still affect the generalizability of the findings. In addition, students who volunteered to participate and who completed the 5 sessions of DWM may have been more motivated to engage with the intervention or more inclined to benefit from mental health resources, which could introduce a selection bias. Moreover, we did not collect data on initial nonrespondents, which prevents us from assessing potential differences between participants and those who did not join the intervention. Second, this study did not include a control group, which limits the ability to draw definitive conclusions about the absolute efficacy of the intervention in this specific population group; furthermore, the lack of randomization may have affected the internal validity of the findings. Third, the reliance on self-reported measures may introduce biases, and the follow-up period may have been too short to assess long-term effects. Finally, the timing of the intervention may have coincided with other academic stressors, influencing participants’ mental health independently. Despite these limitations, the lack of differentiation in the effectiveness of the intervention across sociodemographic and clinical variables suggests that the findings can still be generalized to a wider student audience.

### Conclusions

In conclusion, our findings suggest that the DWM intervention positively impacted students’ mental health by effectively reducing psychological symptoms and enhancing overall well-being. The high levels of acceptability, appropriateness, and feasibility observed in this study show that the program was well received by the student population, indicating its potential for broader implementation. The DWM intervention stands out as a comprehensive and adaptable approach to mental health promotion, effectively catering to diverse individual needs.

## Supplementary material

10.2196/69031 Multimedia Appendix 1 Regression analyses and predicted probabilities of clinical and implementation outcomes.

## Abbreviations

AIM: Acceptability of Intervention Measure
CAMPUS: Characterize and Address Mental health Problems in University Students
*DSM-IV*: *Diagnostic and Statistical Manual of Mental Disorders, Fourth Edition*
DWM: Doing What Matters in Times of Stress
FIM: Feasibility of Intervention Measure
GAD-7: Generalized Anxiety Disorder-7
IAM: Intervention Appropriateness Measure
K-10: Kessler-10
PHQ-9: Patient Health Questionnaire-9
WHO: World Health Organization
WHO-5: World Health Organization-5 Well-Being Index
