# Supplementary material for: Effectiveness of a Self-Guided Digital Intervention for Mental Health and Psychological Well-Being in University Students: Pre- and Postintervention Study
Source: J Med Internet Res. 2025 Aug 19;27:e69031. doi: 10.2196/69031 (PMC12364424; doi:10.2196/69031)

**MULTIMEDIA APPENDIX: Regression analyses and predicted probabilities of clinical and implementation outcomes**

**Table S1. Regression analysis (N=1498)**

|  | **K10_T2** | **95% Confidence Interval** |
| --- | --- | --- |
| K10_baseline | 0.553*** | 0.515,0.591 |
| Age in years | -0.010 | -0.071,0.051 |
| Education, Philosophy and Social Work vs Economics | -0.048 | -0.909,0.812 |
| Law vs Economics | 0.208 | -0.847,1.263 |
| Literature, arts and communication vs Economics | 0.144 | -0.898,1.187 |
| Foreign Languages and Literatures vs Economics | 1.266* | 0.285,2.246 |
| Medicine and Surgery vs Economics | -0.512 | -1.250,0.225 |
| Sciences and Engineering vs Economics | -0.815 | -1.662,0.033 |
| Sport Science vs Economics | -0.121 | -1.271,1.028 |
| Female vs Male | -0.375 | -0.974,0.224 |
| Other Gender vs Male | -2.568 | -9.152,4.017 |
| Years 4-6 or graduand vs First three years | -0.524 | -1.355,0.306 |
| Overtime student vs First three years | 0.383 | -1.885,2.652 |
| Flatmates vs Alone | 0.415 | -1.112,1.942 |
| Parents vs Alone | -0.321 | -1.805,1.163 |
| Other living situation vs Alone | -0.510 | -2.018,0.998 |
| House owned | 0.047 | -0.763,0.858 |
| Constant | 8.287*** | 5.837,10.736 |
| Observations | 1498 | |

Legend: **P*<.05; ** *P*<.01; *** *P*<.001

|  | **PHQ9_T2** | **95% Confidence Interval** |
| --- | --- | --- |
|  |  |  |
| PHQ-9_baseline | 0.569*** | 0.531,0.607 |
| Age in years | -0.009 | -0.054,0.035 |
| Education, Philosophy and Social Work vs Economics | -0.031 | -0.660,0.598 |
| Law vs Economics | 0.169 | -0.604,0.942 |
| Literature, arts and communication vs Economics | 0.624 | -0.138,1.386 |
| Foreign Languages and Literatures vs Economics | 0.767* | 0.053,1.482 |
| Medicine and Surgery vs Economics | -0.160 | -0.697,0.378 |
| Sciences and Engineering vs Economics | -0.152 | -0.769,0.465 |
| Sport Science vs Economics | -0.386 | -1.222,0.449 |
| Female vs Male | -0.379 | -0.813,0.055 |
| Other Gender vs Male | -2.169 | -6.972,2.633 |
| Years 4-6 or graduand vs First three years | -0.399 | -1.005,0.207 |
| Overtime student vs First three years | 0.810 | -0.850,2.471 |
| Flatmates vs Alone | 0.083 | -1.036,1.203 |
| Parents vs Alone | -0.349 | -1.429,0.732 |
| Other living situation vs Alone | -0.468 | -1.570,0.635 |
| House owned | 0.061 | -0.525,0.648 |
| Constant | 2.771** | 1.046,4.496 |
|  |  |  |
| Observations | 1498 |  |

Legend: * *P*<.05; ** *P*<.01; *** *P*<.001

|  | **WHO5_T2** | **95% Confidence Interval** |
| --- | --- | --- |
|  |  |  |
| WHO5_baseline | 0.605*** | 0.563,0.647 |
| Age in years | 0.008 | -0.039,0.056 |
| Education, Philosophy and Social Work vs Economics | -0.078 | -0.763,0.607 |
| Law vs Economics | 0.394 | -0.442,1.231 |
| Literature, arts and communication vs Economics | 0.261 | -0.560,1.082 |
| Foreign Languages and Literatures vs Economics | -0.403 | -1.180,0.375 |
| Medicine and Surgery vs Economics | 0.479 | -0.107,1.064 |
| Sciences and Engineering vs Economics | 0.504 | -0.166,1.173 |
| Sport Science vs Economics | -0.107 | -1.020,0.805 |
| Female vs Male | -0.146 | -0.619,0.326 |
| Other Gender vs Male | -0.334 | -5.569,4.901 |
| Years 4-6 or graduand vs First three years | -0.003 | -0.664,0.658 |
| Overtime student vs First three years | -1.118 | -2.922,0.685 |
| Flatmates vs Alone | -0.323 | -1.536,0.891 |
| Parents vs Alone | -0.094 | -1.266,1.078 |
| Other living situation vs Alone | -0.165 | -1.364,1.034 |
| House owned | 0.046 | -0.589,0.681 |
| Constant | 6.033*** | 4.143,7.923 |
|  |  |  |
| Observations | 1498 |  |

Legend: * *P*<.05; ** *P*<.01; *** *P*<.001

|  | **WHO5_T2** | **95% Confidence Interval** |
| --- | --- | --- |
|  |  |  |
| GAD7_baseline | 0.495*** | 0.457,0.532 |
| Age in years | -0.022 | -0.062,0.017 |
| Education, Philosophy and Social Work vs Economics | -0.090 | -0.650,0.470 |
| Law vs Economics | 0.361 | -0.326,1.047 |
| Literature, arts and communication vs Economics | 0.099 | -0.571,0.770 |
| Foreign Languages and Literatures vs Economics | 0.815* | 0.178,1.452 |
| Medicine and Surgery vs Economics | -0.230 | -0.709,0.249 |
| Sciences and Engineering vs Economics | -0.843** | -1.391,-0.295 |
| Sport Science vs Economics | -0.420 | -1.165,0.324 |
| Female vs Male | -0.206 | -0.598,0.185 |
| Other Gender vs Male | -4.898* | -9.183,-0.613 |
| Years 4-6 or graduand vs First three years | -0.244 | -0.784,0.296 |
| Overtime student vs First three years | 0.032 | -1.442,1.507 |
| Flatmates vs Alone | -0.246 | -1.239,0.746 |
| Parents vs Alone | -0.578 | -1.541,0.386 |
| Other living situation vs Alone | -0.770 | -1.751,0.212 |
| House owned | -0.140 | -0.662,0.382 |
| Constant | 3.908*** | 2.380,5.436 |
|  |  |  |
| Observations | 1498 |  |

Legend: * *P*<.05; ** *P*<.01; *** *P*<.001

**Figure S1. Adjusted predicted probabilities of maximum scores in the acceptability and appropriateness measures by WHO5 score-gender combination**


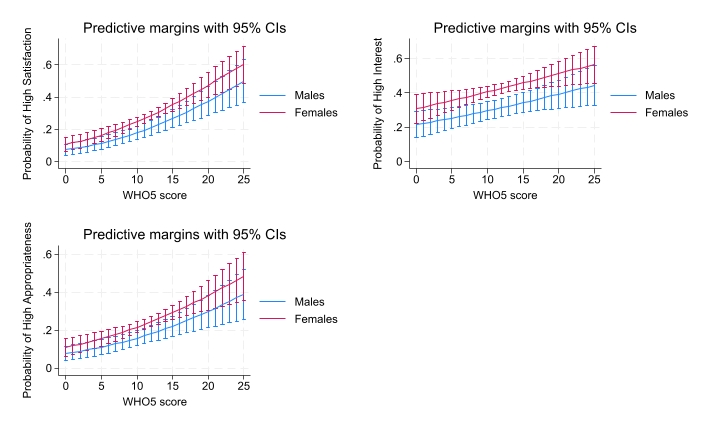

Supplement: Multimedia Appendix 1 [file jmir-v27-e69031-s001.doc]
